# Evaluations of Genomic Prediction and Identification of New Loci for Resistance to Stripe Rust Disease in Wheat (*Triticum aestivum* L.)

**DOI:** 10.3389/fgene.2021.710485

**Published:** 2021-09-28

**Authors:** Vipin Tomar, Guriqbal Singh Dhillon, Daljit Singh, Ravi Prakash Singh, Jesse Poland, Anis Ahmad Chaudhary, Pradeep Kumar Bhati, Arun Kumar Joshi, Uttam Kumar

**Affiliations:** ^1^Borlaug Institute for South Asia, Ludhiana, India; ^2^International Maize and Wheat Improvement Center, New Delhi, India; ^3^Global Wheat Program, International Maize and Wheat Improvement Center (CIMMYT), Texcoco, Mexico; ^4^Department of Biotechnology, Thapar Institute of Engineering and Technology, Patiala, India; ^5^Department of Plant Pathology, Kansas State University, Manhattan, KS, United States; ^6^Department of Biology, College of Science, Imam Mohammad Ibn Saud Islamic University, Riyadh, Saudi Arabia

**Keywords:** GWAS, GS, QTLs, BLUEs, stripe rust, genotyping by sequencing (GBS), *Triticum aestivum* L.

## Abstract

Stripe rust is one of the most destructive diseases of wheat (*Triticum aestivum* L.), caused by *Puccinia striiformis* f. sp. *tritici* (*Pst*), and responsible for significant yield losses worldwide. Single-nucleotide polymorphism (SNP) diagnostic markers were used to identify new sources of resistance at adult plant stage to wheat stripe rust (YR) in 141 CIMMYT advanced bread wheat lines over 3 years in replicated trials at Borlaug Institute for South Asia (BISA), Ludhiana. We performed a genome-wide association study and genomic prediction to aid the genetic gain by accumulating disease resistance alleles. The responses to YR in 141 advanced wheat breeding lines at adult plant stage were used to generate G × E (genotype × environment)-dependent rust scores for prediction and genome-wide association study (GWAS), eliminating variation due to climate and disease pressure changes. The lowest mean prediction accuracies were 0.59 for genomic best linear unbiased prediction (GBLUP) and ridge-regression BLUP (RRBLUP), while the highest mean was 0.63 for extended GBLUP (EGBLUP) and random forest (RF), using 14,563 SNPs and the G × E rust score results. RF and EGBLUP predicted higher accuracies (∼3%) than did GBLUP and RRBLUP. Promising genomic prediction demonstrates the viability and efficacy of improving quantitative rust tolerance. The resistance to YR in these lines was attributed to eight quantitative trait loci (QTLs) using the FarmCPU algorithm. Four (*Q.Yr.bisa-2A.1*, *Q.Yr.bisa-2D*, *Q.Yr.bisa-5B.2*, and *Q.Yr.bisa-7A*) of eight QTLs linked to the diagnostic markers were mapped at unique loci (previously unidentified for *Pst* resistance) and possibly new loci. The statistical evidence of effectiveness and distribution of the new diagnostic markers for the resistance loci would help to develop new stripe rust resistance sources. These diagnostic markers along with previously established markers would be used to create novel DNA biosensor-based microarrays for rapid detection of the resistance loci on large panels upon functional validation of the candidate genes identified in the present study to aid in rapid genetic gain in the future breeding programs.

## Introduction

Wheat is an important cereal crop, providing almost 20% of daily food calories to the world population. With record production of 777 million metric tons (MMT) in the years 2019–2020, and 604 MMT being directly used for human consumption, it cannot still feed the starving section of the population^1^. The demands are yet not met for the growing population despite the increase in the year-by-year production. Besides the deficit in the demand–supply ratio, wheat also faces various serious hindrances in production. Various biotic and abiotic factors hinder the total yield in many ways. *Puccinia striiformis* (*Pst*) causing yellow rust or stripe rust (YR) in wheat is causing significant economic damages to the production of wheat, causing losses up to 70% of total yields in epidemic conditions (Chen, 2005). Sustainable management of wheat stripe rust is only possible by identifying and introducing rust resistance genes into the wheat elite cultivars.

The quantitative nature of many diseases coupled with lack of knowledge of pathosystems and dynamics of host genotypic stability across environments makes it imperative to study the role of environment and genotype-by-environment interaction (G × E). Breeding programs are often affected by environment due to the variable response of host–pathogen interactions, thus deluding identification of genotypes stable for disease resistance (Das et al., 2019). Hence, any further evaluation of the genotypes for identification of loci of resistance via linkage mapping or association mapping or even prediction studies is highly influenced with the unstable phenotypic response. Various stability approaches have been designed and implemented across crops for several phenotypes (including diseases) to overcome the variance introduced by G × E (Juliana et al., 2017; Tekalign et al., 2017; Elbasyoni et al., 2019; Parmley et al., 2019; Jiwuba et al., 2020).

Genomic prediction (GP) models are developed using the phenotypic and genotypic information calculating marker effects, which are further used for calculating genomic estimated breeding values (GEBVs) (Azizinia et al., 2020). With an assumption of markers being in linkage disequilibrium (LD), each marker’s effect regardless of significance is used to capture genetic variance (Crossa et al., 2017; Robertsen et al., 2019). Training the models on a training set and utilizing it for calculating GEBVs of a test set help in calculating the prediction accuracies/ability of the model as cross-validation (CV). The CV is calculated as a correlation of GEBVs and the phenotypic values of the training set. Various GP statistical models with high accuracy used in many crops for different traits are primarily of three types: linear parametric methods such as genomic best linear unbiased prediction (GBLUP), extended GBLUP (EGBLUP), and ridge-regression BLUP (RRBLUP); nonlinear semi-parametric such as Reproducing Kernel Hilbert Space (RKHS); and nonlinear and nonparametric methods such as random forest (RF) (Crossa et al., 2017; Wang et al., 2018). Genomic selection (GS) along with the rapid generation of advancement through speed breeding (Watson et al., 2018) could be beneficial leading to higher selection intensity over a smaller period, which could further increase rates of genetic gains. As established by Azizinia et al. (2020), implementation of GS models in rust resistance breeding has been studied in different wheat breeding programs (Rutkoski et al., 2011, 2014, 2015a, b; Ornella et al., 2012; Daetwyler et al., 2014; Juliana et al., 2017; Muleta et al., 2017).

The availability of draft sequences in wheat has become a useful resource due to a well-annotated and high-quality reference genome (Appels et al., 2018). Genome-wide association studies (GWASs) has various advantages over traditional quantitative trait locus (QTL) mapping and linkage analysis, for instance, comprehensive allele coverage, which is economical, rapid, and the most important (Olukolu et al., 2016). GWAS can exploit LD and detect loci to observe marker–trait associations (MTAs) and find novel genes linked with aggregate quantitative phenotypic traits (Yang et al., 2015; Liu et al., 2017). High LD in wheat can considerably decrease the markers required for detecting MTAs (Chao et al., 2010). GWAS has been used for mapping loci for various disease resistance in wheat such as leaf rust, stem rust, and spot blotch (Gao et al., 2016; Kankwatsa et al., 2017; Edae et al., 2018; Kaur et al., 2021; Tomar et al., 2021). Besides, GWAS has enabled verification of stripe rust resistance and identification of the underlying resistance genes in wheat (Juliana et al., 2017, 2018). GWAS and GP-based breeding programs have substantially accelerated the detection of various trait-related genomic regions with high accuracy and further use in developing and selecting new elite cultivars.

Only a few GP studies have been conducted for rust resistance, and among those significantly few are based on RF model, while none of the GP studies has been reported based on the Indian wheat scenario. In the present study, a collection of advanced breeding lines from CIMMYT was used to detect the MTAs and GP of rust resistance. The current study goals were to (1) compare the nonparametric method with some of the other the well-known models widely applied in GP for predicting wheat rust disease, (2) detect genomic regions responsible for resistance against stripe rust disease, and (3) shape the possible ground work for DNA-based biosensors for pathogen detection for precision agriculture applications.

## Materials and Methods

### Plant Genetic Material and Screening for Rust Resistance

A set of 141 advanced spring wheat breeding lines developed by the global wheat program of CIMMYT Mexico and sent to South Asia were used in this study (Supplementary Table 1). The lines were planted at BISA Ludhiana for three consecutive years (E1 = 2017, E2 = 2018, and E3 = 2019) in two replications. Each genotype was planted in a 4-m-long plot of six rows with a row-to-row distance of 22 cm. The spreader rows of susceptible genotypes, namely, Agra Local, PBW343, and HD2967, were planted on both sides of plots as borders. Indian Institute of Wheat and Barley Research, Regional Station, Shimla, India, provided the mixture of YR races, viz., 78S84, 46S119, 110S119, and 238S119 (Supplementary Table 4; Singh et al., 2020; Arora et al., 2021). The seedlings were raised in plastic trays in the glasshouse and inoculated with the mixture of YR races using the urediospore–talc mixture to multiply the pathogen spores. The glasshouse’s temperature was maintained below 20°C with 100% relative humidity to aid in infection. The spores were collected from the infected plants to inoculate the genotypes in the field during the first and second weeks of January. The border rows and the population were inoculated with a liquid inoculum containing approximately 1 g of stripe rust spores suspended in 10 L of water with two to three drops of Tween 20 as a dispersant solution. The disease was evaluated according to Line and Qayoum (1992) as a percentage of diseased areas covering flag leaves on a 0–100% scale.

To limit the number of escapes, stripe rust response was evaluated twice during the mid-phase to advanced phase of disease development from adult plants when the spreader rows showed complete susceptibility. These evaluations were performed between plant heading (GS50, growth stage 50 on the Zadoks scale) and grain filling stage (GS80) (Zadoks et al., 1974). Only the reaction showing the highest disease severity between the two stages (usually the later) was used in further analysis.

### Genotyping for Stripe Rust

Seeds of all lines were obtained from the CIMMYT genetic resources program, and genomic DNA was extracted from five bulked leaves using a modified cetyltrimethylammonium bromide (CTAB) procedure as described by Dreisigacker et al. (2016) in CIMMYT molecular protocol manual, Mexico. The DNA samples were sent to Kansas State University, United States, for genotyping by sequencing (GBS). GBS was performed following the protocol described by Poland et al. (2012). All lines were sequenced with Illumina HiSeq2000. GBS–single-nucleotide polymorphism (GBS-SNP) markers were called with TASSEL v5.2 pipeline GBSv2 (Bradbury et al., 2007) and aligned to the reference Chinese Spring Wheat Assembly (RefSeq v1.0). The raw SNP files generated by the GBSv2 pipeline were filtered removing SNPs with greater than 30% missing data, 20% heterozygosity, and minor allele frequency of less than 5%. The resultant 14,563 SNP markers were subjected to data imputation using Beagle v4.1 (Browning and Browning, 2016). Genotyping Hapmap file is provided as a supplementary file (Supplementary Table 2).

### Statistical Analysis

The analysis of variance (ANOVA) of three seasons and replicated data was done using META-R version 6.4 (Alvarado et al., 2020). The adjusted means of replicates combined across the environments in the trial were obtained by fitting mixed linear models (MLMs) using the equation:


$$Y_{ijk}=\mu +E_{j}+R_{i}\left(E_{j}\right)+G_{k}+E_{j}XG_{k}+\epsilon_{ijk}$$

where *Y*_*ijk*_ is the trait of interest, *μ* is the mean effect, *E*_*j*_ is the *j*th environment, *R*_*i*_(*E*_*j*_) is the effect of the *i*th replicate in the *j*th environment, *G*_*k*_ is the effect of the *k*th genotype, *E*_*j*_*XG*_*k*_ is the effect of the *j*th environment by *k*th genotype interaction, and *ϵ*_*ijk*_ is the error associated with the *i*th replication, the *j*th environment, and the *k*th genotype, which is assumed to be normally and independently distributed, with mean zero and homoscedastic variance σ^2^. The genotypes and replications were selected as fixed effects, while environments were selected as random effects to calculate adjusted means across different environments’ replicates. Further principal component analysis (PCA) and biplot analysis were performed, using FactoMineR and factoextra libraries in R, to identify the relationship between the environment and rust scores. The broad-sense heritability was estimated using the formula,


$$H^{2}=\frac{\sigma_{g}^{2}}{\sigma_{g}^{2}+\sigma_{ge}^{2}/nenv+\sigma_{e}^{2}/\left(nrepsXnenv\right)}$$

where $\sigma_{g}^{2}$ is genotypic variance, $\sigma_{ge}^{2}$ is G × E variance and $\sigma_{e}^{2}$ is error variance, *nenv* is the number of environments, and *nreps* is the number of replications. The broad-sense heritability estimated the quality of a breeding program for the traits and the environments. The least significant difference (LSD) with type I error, α = 0.05 of the level of significance, was calculated using the formula,


$$LSD=t_{\left(1-0.05,dferror\right)}\times ASED$$

where *t* is the cumulative Student’s *t*-test distribution, *dferror* is the degrees of freedom for the variance of error, and ASED is the average standard error of the differences between pairs of means. And the coefficient of variation is calculated using the formula,


$$CV=100\times \frac{ASED}{grandmean}$$

### Genomic Prediction Models

In the present study, five statistical algorithms were used for GP, RRBLUP (Meuwissen et al., 2001), GBLUP (VanRaden, 2008), EGBLUP (Jiang and Reif, 2015), RKHS (Gianola and Van Kaam, 2008; De Los Campos et al., 2010), and RF (Breiman et al., 2001). GP was performed using GBLUP, RRBLUP, EGBLUP, RKHS, and RF models from G × E data in the BWGS pipeline (Charmet et al., 2020). GBLUP is a modification of the conventional BLUP used for predictions by estimating line effects, where genomic relationships are used instead of the traditional pedigree relationships. GBLUP fits individuals as random effects, and the covariance among individuals is given by G estimated from genome-wide markers calculating genomic-kinship matrix. With the use of mixed-model solver of RRBLUP library, restricted maximum-likelihood is calculated using the formula,


y=Xβ+Zμ+ϵ

where *Xβ* is the mean with *β* as a fixed-effect vector and *μ* as a random-effect vector of additive genetic effects with variance for $\mu =K\sigma_{\mu }^{2}$. The residual variance for $\epsilon =I\sigma_{\epsilon }^{2}$. It has is a single variance component other than the residual error and is close to ridge regression (ridge parameter $\lambda =\sigma_{\mu }^{2}/\sigma_{\epsilon }^{2}$). RRBLUP is a mixed GP primary model using package glmnet (Friedman et al., 2010) and, in theory, equivalent to GBLUP with a smoothening parameter λ as ratio of variance components. EGBLUP model is BLUP using a “squared” relationship matrix to model epistatic 2 × 2 interactions, as described by Jiang and Reif (2015) using the BGLR library. Linear models uses information from relatives for breeding value prediction, with closer relative information weighted more heavily. These models assume that the trait is conditioned by an infinitesimal number of additive loci (no major genes).

RKHS model is based on genetic distance and a kernel function to regulate the distribution of marker effects using multiple linear regression enabling it to fit for complex interaction patterns (Gianola and Van Kaam, 2008; De Los Campos et al., 2010) with an ability to account for non-additive effects with regressions in a higher-dimensional space using the BGLR library. Thus, the RKHS method is suitable even for detecting non-additive effects. RF model is based on RF regression, using randomForest library (Breiman et al., 2001). In the RF model, a series of regression trees were grown independently to the largest extent possible using subsets of bootstrap samples. At each split of the tree, a random subset of variables is selected to identify the best split. RF is able to capture interactions between markers.

### Cross-Validation Scheme

A five-fold CV scheme (Owens et al., 2014) was performed by randomly dividing the advanced breeding lines into approximately equal subsets/folds. The procedure was repeated 1,000 times, and the resulting accuracy was averaged. CV based on prediction accuracies has previously been used to evaluate GP models (Bernardo and Yu, 2007; Heffner et al., 2009; Crossa et al., 2010; Jannink et al., 2010; Iwata and Jannink, 2011). Prediction accuracy of each fold was calculated as Pearson’s correlation of observed values, i.e., best linear unbiased estimates (BLUEs) to the predicted values or genomic estimated breeding values (GEBVs). The average of the five-fold correlations was calculated as the prediction accuracy of the iteration. All calculations were performed in R 4.0.2 (R Core Team, 2019).

### Marker–Trait Associations Analysis and False Discovery Rate Correction

The FarmCPU algorithm of GAPIT 3.0 (Wang and Zhang, 2021) was used for 141 genotypes with 14,563 SNP markers for the association study. False discovery rate (FDR)-adjusted *p*-values (0.05) from GAPIT were used for significant association threshold (Benjamini and Hochberg, 1995). Significant MTAs from the GWAS result were visualized by an integrated display of MTAs, gene structure, and LD matrix in the IntAssoPlot package (He et al., 2020) in R 4.0.2 (R Core Team, 2019).

### Validation of Significantly Associated Single-Nucleotide Polymorphisms and the Postulation of Candidate Genes

Significant SNPs associated with stripe rust from GWAS results of the present study were further analyzed if the MTAs fall within the proximity of genomic regions known for rust resistance using functional annotation from the reference genome assembly (IWGSC RefSeq v1.0) and compared with known QTLs/genes. Functional annotation of the genes in the genomic regions harboring significant SNPs were retrieved and examined for their association with yellow rust resistance. Subsequently, genes’ annotated protein functions were literature mined to establish these genes’ relationship with disease resistance. The IntAssoPlot package (He et al., 2020) was used to calculate the extent of linkage between markers from the D prime (D’) estimates and to visualize the LD patterns.

## Results

### Phenotypic Variability and Estimates of Heritability

**FIGURE 1:**
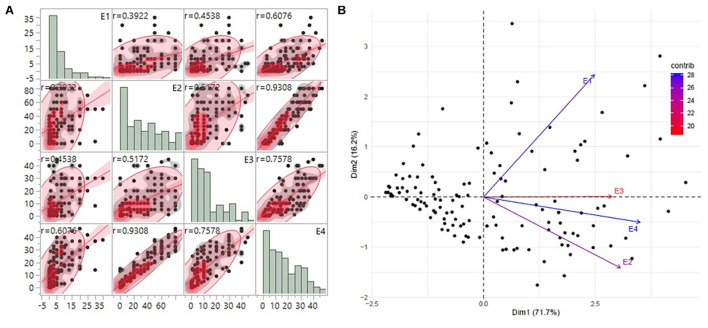
**(A)** The trends of disease score distribution for 141 advanced breeding lines across the three individual environments E1, E2, and E3 along with the G × E data as E4. The histograms show the disease distribution across the environments, while the dot plots show the correlation of disease for respective lines across the environments. **(B)** PCA biplot of disease score across the four environments using the first and second principal component vectors. PCA, principal component analysis.

Substantial variation was observed among genotypes for rust disease. However, as seen from the histogram, the distribution was skewed toward resistance. The majority of genotypes showed high-to-moderate resistance to disease reaction, and only a few genotypes showed high susceptibility (Figure 1A). Although there was consistency in the resistance reaction over the years, however, to enhance accuracy in the results, the G × E model was fitted to obtain the disease’s average effect on the different lines across the years (Supplementary Table 3). This combined G × E disease severity data were used as environment 4 (E4), which showed a strong positive correlation from 0.60 to 0.93 with all the three environments. The disease score of E4 was successfully able to explain the across year disease variability, which would eliminate the year-wise variability and provides significant stable data for both GWAS and GP studies (Figure 1B). Moderate heritability was observed 0.57 for G × E with a noteworthy genotypic significance of 1.7E-09 for genotypes as random effects, while 4.51E–13 was observed for genotypes as fixed effects (Table 1).

**TABLE 1:** Combined analysis of variance of 141 advanced lines evaluated for stripe rust disease resistance for disease severity recorded for three environments.

Statistics	BLUPs	BLUEs
Heritability	0.573759	
Genotype variance	85.74016	
Gen × Loc variance	167.2694	
Residual variance	47.63594	47.63593
Grand mean	15.65485	15.65485
LSD	16.82923	22.21773
CV	44.08781	44.08781
Replicates	2	2
Environments	3	3
Genotype significance	1.7E-09	4.51E-13

*LSD, least significant different; CV, coefficient of variation; BLUP, best linear unbiased prediction; BLUE, best linear unbiased estimate.*

### Evaluation of Statistical Models for Prediction Accuracy

**FIGURE 2:**
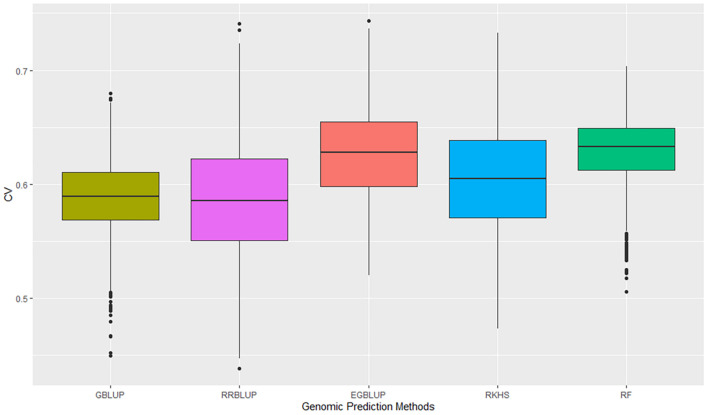
Prediction accuracies of five genomic prediction methods for the G × E data calculated as correlation of GEBVs predicted to phenotypic data of the advanced breeding lines. GEBVs, genomic estimated breeding values.

The GP was performed in a five-fold scheme of dividing the 141 lines randomly into five subgroups. The average prediction accuracy were 0.59 for GBLUP, 0.59 for RRBLUP, 0.60 for RKHS, 0.63 for the EGBLUP, and 0.63 for the RF model (Figure 2 and Table 2). The moderately high range of prediction accuracy of all the models suggests that the models can predict the GEBVs for stripe rust resistance. Despite EGBLUP and RF models showing similar CVs (>3% than other models), the interquartile range (IQR) represented by RF (0.037) was smaller than that of EGBLUP (0.042), indicating more stability of the model. The RF model was found to be the most efficient method among all the techniques, due to higher median prediction accuracy of 0.633 along with lowest standard variation and IQR.

**TABLE 2:** Prediction accuracies using five-fold cross-validation for different genomic prediction models for the G × E data of stripe rust disease score as E4.

	GBLUP	EGBLUP	RRBLUP	RKHS	RF
Mean	0.588	0.627	0.586	0.605	0.628
Std. Dev.	0.033	0.040	0.050	0.048	0.032
Min	0.449	0.520	0.438	0.473	0.505
Median	0.589	0.628	0.585	0.605	0.633
Max	0.680	0.743	0.741	0.733	0.703
IQR	0.042	0.057	0.072	0.068	0.037

*Std. Dev., standard deviation; min, minimum prediction accuracy; max, maximum prediction accuracy; IQR, interquartile range; GBLUP, genomic best linear unbiased prediction; EGBLUP, extended genomic best linear unbiased prediction; RRBLUP, ridge-regression best linear unbiased prediction; RKHS, reproducing kernel Hilbert space; RF, random forest; IQR, interquartile range.*

### Genome-Wide Association Mapping of Resistance to Stripe Rust at the Adult Plant Stage

**FIGURE 3:**
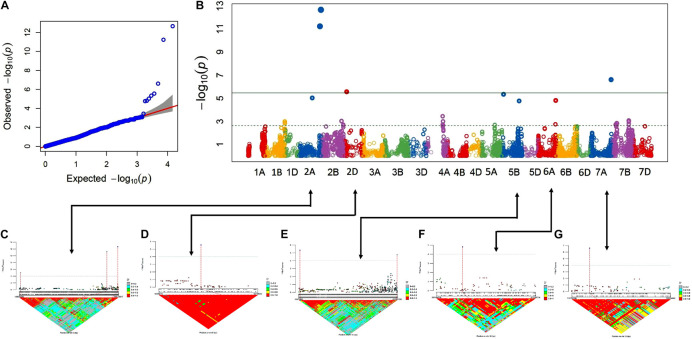
GWAS results for stripe rust resistance. **(A,B)** Q-Q and Manhattan plots of SNPs associated with stripe rust resistance. Horizontal line depicts significance threshold level. **(C–G)** Manhattan plots of SNP-specific genomic region clusters on chromosomes 2A, 2D, 5B, 6A, and 7A and corresponding linkage disequilibrium (LD) *D*′ patterns. SNP names and corresponding local LD value patterns are in the bottom part of the graph. Numbers within the diamonds of the triangular LD matrix are *D*′ values. GWAS, genome-wide association study; SNPs, single-nucleotide polymorphisms.

**FIGURE 4:**
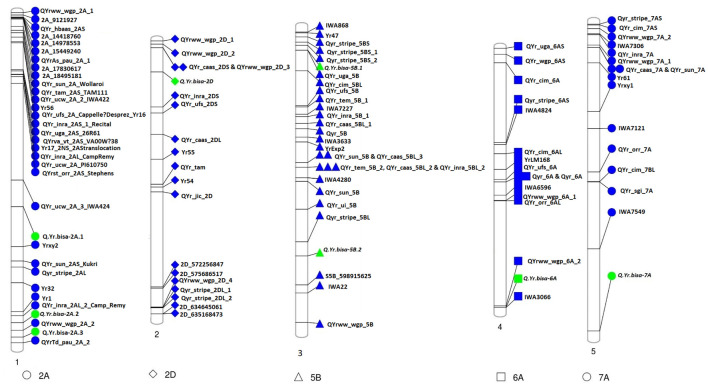
Physical map of candidate QTLs for stripe rust resistance (represented in green) indicating physical positions on the respective chromosomes along with genes/QTLs identified in previous studies (represented in blue). QTLs, quantitative trait loci.

The association analysis was performed using the FarmCPU algorithm with 14,563 SNP and G × E disease severity data. A total of eight MTAs (eight QTLs) showed significant associations with stripe rust resistance at five different chromosomes (2A, 2D, 5B, 6A, and 7A). The phenotypic effect explained by these QTLs was in the range of 2.74–5.5% with both major and minor alleles imparting resistance to the lines used in this study. The Manhattan plots, gene locations, and LD haplotypes of genomic regions with significant MTAs are given in Figure 3. The markers significantly associated with stripe rust, their chromosomal locations, *p*-values, the closest mapped gene(s), and predicted function in the *Triticum aestivum* gene transcripts are summarized in Tables 3, 4. The significance of the MTAs with FDR-adjusted *p*-value was highly significant for *Q.Yr.bisa.2A.3*. The genes flanking to the significant QTLs were identified, and the functions of the encoded proteins of these genes have been provided in Table 4. Furthermore, we compared the detected QTLs with previously known QTL/genomic regions on a physical map; from the literature mining, four QTLs appeared to be potentially novel (Figure 4). The identified QTLs were associated with the disease resistance-related protein family (Table 4).

**TABLE 3:** Summary of the QTL mapping using GWAS in the present study.

QTLs	SNP marker	Alleles	Effective allele	Chr	Pos. (Mb)	MAF	FDR Adj. *p*-val	Effect	*p*-Val (-log10)
*Q.Yr.bisa-2A.1*	S2A_451543170	C/T	C	2A	451.543	0.099	0.022855184	5.114	5.0261
*Q.Yr.bisa-2A.2*	S2A_719332307	G/T	T	2A	719.332	0.163	0.000000043	–5.491	11.2247
*Q.Yr.bisa-2A.3*	S2A_753261678	A/C	C	2A	753.262	0.422	0.000000003	–5.526	12.6547
*Q.Yr.bisa-2D*	S2D_76635286	C/T	C	2D	76.635	0.301	0.010022488	3.518	5.5602
*Q.Yr.bisa-5B.1*	S5B_56640410	A/T	T	5B	56.640	0.124	0.013329820	–3.595	5.3395
*Q.Yr.bisa-5B.2*	S5B_598915625	A/C	A	5B	598.916	0.213	0.031241407	3.039	4.7654
*Q.Yr.bisa-6A*	S6A_602916015	C/G	G	6A	602.916	0.351	0.031241407	–2.742	4.8066
*Q.Yr.bisa-7A*	S7A_717077502	C/T	C	7A	717.078	0.408	0.001195850	3.728	6.6085

*Chr, chromosome; Mb, million bases; MAF, minor allele frequency; QTL, quantitative trait locus; GWAS, genome-wide association study; SNP, single-nucleotide polymorphism; FDR, false discovery rate.*

**TABLE 4:** Summary of genes found adjacent to SNPs associated with the QTLs mapped in the present study based on the gene annotation using wheat reference sequence (RefSeq v1.0) annotation database.

QTL	Chr	Dist. from SNP (bp)	Genes IDs	Annotation
*Q.Yr.bisa-2A.1*	2A	−525,831	*TraesCS2A01G274900*	Importin-like protein
		10,809	*TraesCS2A01G275000*	Eukaryotic aspartyl protease family protein
*Q.Yr.bisa-2A.2*	2A	−13,141	*TraesCS2A01G483500*	Organic cation transporter protein
		11,815	*TraesCS2A01G483600*	Elongation factor 1-alpha
*Q.Yr.bisa-2A.3*	2A	−6,179	*TraesCS2A01G543100*	Acyl-protein thioesterase 1
		6,259	*TraesCS2A01G543200*	Leucine-rich repeat receptor-like protein kinase
*Q.Yr.bisa-2D*	2D	−857	*TraesCS2D01G131100*	Mediator of RNA polymerase II transcription subunit 3
		4,259	*TraesCS2D01G131200*	ATP-dependent RNA helicase DDX47
*Q.Yr.bisa-5B.1*	5B	−77,352	*TraesCS5B01G051900*	Cullin-associated NEDD8-dissociated protein 1
		338,835	*TraesCS5B01G052000*	Patatin
*Q.Yr.bisa-5B.2*	5B	−161,555	*TraesCS5B01G422900*	ATP-dependent Clp protease ATP-binding subunit
		205,384	*TraesCS5B01G423000*	ATP-dependent Clp protease ATP-binding subunit
*Q.Yr.bisa-6A*	6A	−2,481	*TraesCS6A01G384600*	Fatty acid hydroxylase superfamily protein
		129,242	*TraesCS6A01G384700*	Protein kinase family protein
*Q.Yr.bisa-7A*	6A	−7,604	*TraesCS7A01G539900*	Receptor-like protein kinase
		3,441	*TraesCS7A01G540000*	Mediator of RNA polymerase ii transcription subunit 15a

*Negative sign shows that the gene was present upstream of the associated SNP, and positive sign shows that the gene was present downstream of the associated SNP. QTL, quantitative trait locus; Chr, chromosome; SNP, single-nucleotide polymorphism.*

### Linkage Disequilibrium-Based Linked Quantitative Trait Loci

The LD of the eight MTAs on 2A, 2D, 5B, 6A, and 7A chromosomes was obtained and used to demarcate as LD-based QTL (Figures 3A–G). For LD estimation, the eight significant MTAs associated with YR resistance in advanced wheat breeding lines were used. Marker pairs with a *D*′ value greater than 0.80 and the *p*-value for the existence of LD equal to 0 were designated into an LD-based QTL. The eight significant MTAs, as well as their chromosomal position and designated QTLs, are shown in Table 3. Manhattan plots represent the information associated with significant loci and LD and linkage to the significant loci’s in the genomic assembly. Linking lines with the associated markers with LD of the region were also highlighted in systematics schemes.

### Allelic Effect of Identified Quantitative Trait Loci for Stripe Rust

**FIGURE 5:**
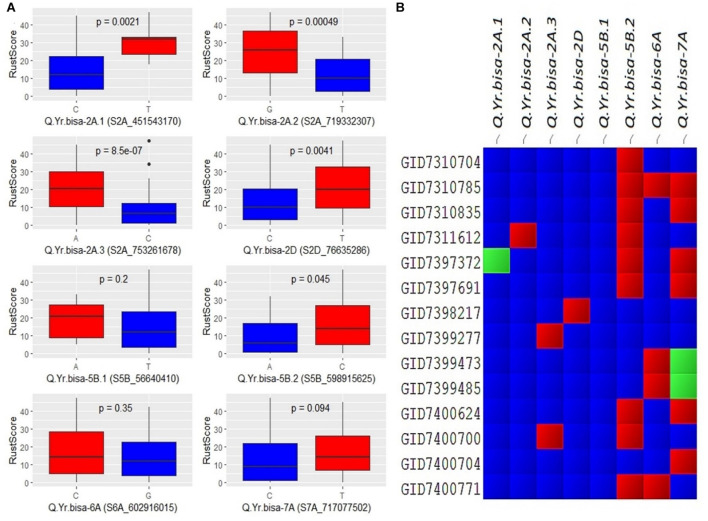
**(A)** Boxplots showing the effect of phenotypic variation between the two alleles of the associated SNPs of identified QTLs for stripe rust disease score; the Kruskal–Wallis test was used to determine the significant differences between the mean values of two alleles. **(B)** Cell plot of the distribution of the different alleles of significant SNPs of identified QTLs in the totally resistant lines from the panel. ^#^Blue color indicates presence of resistant allele, red color indicates presence of susceptible allele, and green color indicates presence of heterozygous allele. SNPs, single-nucleotide polymorphisms; QTLs, quantitative trait loci.

The allelic effect of disease severity between the two alleles of the SNPs was plotted for all eight significant QTLs (Figure 5). The biallelic effects of the QTLs showed distinctive variation between the two alleles. Most of the alleles had a significant impact, indicating a larger phenotypic effect by the resistance allele’s presence. For example, the minor allele of *Q.Yr.bisa-2A.3*, i.e., C allele, showed a significant effect on disease resistance with the highly significant difference among the two alleles. Similarly, the major allele C of *Q.Yr.bisa-2D* significantly contributed to resistance. For *Q.Yr.bisa-6A*, the allele G was associated with resistance to stripe rust. The significant difference among the alleles C and T of *Q.Yr.bisa-7A* was significantly less between the two alleles with resistance associated with the C allele. This outcome suggests that even though all alleles were associated with the stripe rust resistance in the set of genotypes used, the 2A and 2D genomic regions were highly responsible for resistance in the panel (Figure 5A). The presence of multiple genomic regions for stripe rust resistance in the panel with varying effects suggests the quantitative nature of the genes involved. The presence or absence of the resistant alleles in the highly resistant lines with a disease severity score of zero leads to identifying the QTL composition of the 14 resistant breeding lines. Four (GID7310704, GID7398217, GID7399277, and GID7400704) advanced breeding lines were identified to have seven of the eight identified QTLs from the present study (Figure 5B). These lines are the primary targets for transfer of these QTLs in future breeding programs.

## Discussion

The problem of population explosion can only be dealt with by increasing the total production and yields for important cereal crops to feed the growing population. This situation is further made complex by the constant threat of various biotic stresses. The breeding programs must continuously introduce new sources of disease resistance to counter the ever-evolving pathogen. Wheat stripe rust is one of the critical economically significant diseases that threaten the wheat production. The current study was planned to identify new disease resistance sources, using advanced breeding lines from CIMMYT, Mexico, against the stripe rust pathogens prevalent in South Asia.

### Effect of Reference and Validation of Population on Genomic Selection

We estimated the GP accuracy using five models for stripe rust disease. The stripe rust disease resistance in wheat is controlled by many small- to large-effect QTLs/genes. Instead of relying on single to few major effect loci, GS helps to include many loci that are spread across the genome with higher resolution and power (Jannink et al., 2010). As evident from the results, EGBLUP and the RF models could predict GEBVs with moderately high accuracy. Besides the RF model having higher prediction accuracies among all models, the GEBVs predicted by RF model also showed lower standard deviations and CV. CV based on prediction accuracies explains the efficiency of the GS models as advocated in various earlier studies in plants (Bernardo and Yu, 2007; Heffner et al., 2009; Crossa et al., 2010; Jannink et al., 2010; Iwata and Jannink, 2011).

Prediction accuracies are reasonably high under homogenous breeding lines, but it is far from practice. Introducing new lines over the years and phenotyping on multiple locations are critical to the accomplishment of GS to improve resistance cultivars. Among the 141 lines that were randomly selected as the training population, and the testing population, the highest prediction accuracy of 0.628 was observed for the RF model followed by 0.627 of EGBLUP model. Since these testing populations were advanced screened resistant lines, moderately high prediction accuracy was observed with higher disease pressure. No study reported the use of GP for stripe rust disease for wheat in India. The present study provides an essential precursor in utilizing the GS for breeding stripe rust and other disease-resistant cultivars.

### Candidate Resistance-Associated Loci Identified by Genome-Wide Association Study

The high-density SNPs provided insights into the functional causal variant(s) underlying YR resistance. We identified eight significant MTAs using GWAS and analyzed the LD of the candidate regions containing significant SNPs. Furthermore, we extracted information for the candidate genes, including their possible functional roles in disease resistance. The genes identified were directly or indirectly involved in pathogenesis or plant–pathogen interaction (Table 4). In the present study, the FarmCPU algorithm identified eight significant MTAs associated with stripe rust on chromosomes 2A, 2D, 5B, 6A, and 7A. *Q.Yr.bisa-2A.3* on chromosome 2A at the physical position 753.3 Mb identified for YR resistance in this study was found in the vicinity of a *Q.YrTd.pau.2A.2* mapped at 766.16 Mb by Dhillon et al. (2020). Another QTL mapped on chr2D (*Q.Yr.bisa-2D*) at a terminal end of 2DS was identified in the vicinity (∼70 Mb apart) of *QYr.caas-2DS_Libellula* (Lu et al., 2009). The *Q.Yr.bisa-6A* at 602.91 Mb at chromosome 6A was mapped in a region, where IWA3066 marker has been identified to be associated with YR rust-resistant by Maccaferri et al. (2015) in the same genomic region. However, the study involved used linkage mapping instead of physical mapping. *Q.Yr.bisa-7A* on chromosome 7A at 717.1-Mb position in the present study is likely to be a new stripe rust resistance locus, as we were not able to literature mine any gene/QTL linked to stripe resistance mapped in this region.

### Potential Candidate Resistance Genes for the Significant Stripe Rust Quantitative Trait Loci

*Q.Yr.bisa-2A.1* was found adjacent to *TraesCS2A01G274900* encoding Importin-like protein and *TraesCS2A01G275000* encoding aspartyl protease family protein. Importin from *Arabidopsis thaliana* is a nuclear import receptor (Smith et al., 1997), and encoding aspartyl protease family protein regulates defense responses in *A. thaliana* (Xia et al., 2004). *Q.Yr.bisa-2A.2* is found adjacent to *TraesCS2A01G483500* encoding Organic cation transporter protein and *TraesCS2A01G483600* encoding Elongation factor 1-alpha (EF-1α). Organic cation transporter protein affects root length responses to cadaverine in *Arabidopsis* (Strohm et al., 2015). EF-1α has been identified as being *Fusarium*-responsive in wheat (Kruger et al., 2002; Han et al., 2005) and interrelated with numerous biological processes including senescence and tissue longevity (Silar and Picard, 1994). The *Q.Yr.bisa-2A.3* was found to be associated with the candidate genes *TraesCS2A01G543100*, encoding Acyl-protein thioesterase 1, and *TraesCS2A01G543200* encoding Leucine-rich repeat receptor-like protein kinase (PK). Acyl-protein thioesterase 1 is key regulatory enzyme for dynamic palmitoylation (Garland et al., 2018). Leucine-rich repeat receptor-like PK is well known to play a role in plant defense and associated with stripe rust resistance (Zhang et al., 2019). It indicates that *TraesCS2A01G543200* may be the presumably aspirant resistance gene, while the role of *TraesCS2A01G543100* could not be overlooked. Because resistance genes in plants are normally assembled in clusters, some may play immediate imperative roles (Zhao et al., 2016; Kourelis and Van Der Hoorn, 2018). Hence, the characterization of these genes by molecular approaches may reveal additional channels of rust resistance in wheat.

The *Q.Yr.bisa-2D* was found to associate with *TraesCS2D01G131100*, a mediator of RNA polymerase II transcription subunit 3, and with *TraesCS2D01G131200*, which encodes ATP-dependent RNA helicase. RNA polymerase II transcription subunit 3 is involved at RNA polymerase II transcriptive specificity of wheat leaves at the early stages of rust infection (Pure et al., 1984). ATP-dependent RNA helicase has been reported to be critical for pre-mRNA splicing, cold-responsive gene regulation, and cold tolerance in *Arabidopsis* (Guan et al., 2013). *Q.Yr.bisa-5B.1* was associated with genes *TraesCS5B01G051900* encoding Cullin-associated NEDD8-dissociated protein 1 and *TraesCS5B01G052000*, which encodes patatin. Cullin-associated NEDD8-dissociated protein 1 regulates plant height, flowering time, seed germination, and root architecture in tomato (Cheng et al., 2020); and the *Arabidopsis* patatin-like protein 2 (PLP2) plays a role in cell death execution and differentially affects the biosynthesis of oxylipins and resistance to pathogens (La Camera et al., 2009). The *Q.Yr.bisa-5B.2* was found in-between *TraesCS5B01G422900* and *TraesCS5B01G423000*, both the gene encoding ATP-dependent Clp protease ATP-binding subunit. ATP-dependent Clp protease ATP-binding subunit responds to cold stress in rice seedlings (Cui et al., 2005). Its levels significantly increased during cadmium stress in tobacco and *Amaranthus hybridus* L. roots (Xie et al., 2014; Jin et al., 2016).

The *Q.Yr.bisa-6A* was found to be associated with *TraesCS6A01G384600* encoding fatty acid hydroxylase superfamily protein and *TraesCS6A01G384700* encoding PK family protein gene. Fatty acid hydroxylase superfamily protein is essential for sporopollenin synthesis in *Arabidopsis* pollen (Dobritsa et al., 2009). PK family protein gene confers temperature-dependent resistance to wheat stripe rust (Fu et al., 2009). PKs are vital for transmembrane signaling, regulating plant development and adaptation to various environments (Liang and Zhou, 2018). Numerous kinase proteins have been linked to plant innate immunity. For example, a kinase and a putative START lipid-binding domain are essential to confer wheat rust resistance of *Yr36* (Fu et al., 2009). Stripe rust resistance gene *Yr15* (WTK1) (Klymiuk et al., 2018) and barley (*Hordeum vulgare* L.) stem rust (*Puccinia graminis* f. sp. *tritici*) resistance gene *Rpg1* (Brueggeman et al., 2002) contain a structure with tandem kinase domains. The *Q.Yr.bisa-7A* was associated with *TraesCS7A01G539900*, which encodes receptor-like PK gene conferring temperature-dependent resistance to wheat stripe rust (Fu et al., 2009) and *TraesCS7A01G540000* encoding mediator of RNA polymerase II transcription subunit 15a, which is known to negatively regulate disease resistance against stripe rust (Maytalman et al., 2013). Furthermore, the SNP markers associated with QTLs could be developed into PCR markers, which can be utilized for marker-assisted selection in stripe rust resistance breeding programs and further could be used to develop an electrochemical-based DNA platform for nucleic acid for rapid detection applications, Further, this may serve as the foundation for an efficient, responsive, and low-cost framework.

## Conclusion

The high accuracy percentage from the GP models indicates that the stripe rust resistance trait does not present complicating modeling factors such as the presence of great dominance and epistasis. The RF model based on ML is flexible and does not depend on an *a priori* specification adjustment of the model, making it easier to contemplate such complicated factors. Evaluations of the accuracy of the stripe rust resistance prediction confirmed that both EGBLUP and RF models showed better effectiveness, incurring an even lower computational rate. Four (*Q.Yr.bisa-2A.1*, *Q.Yr.bisa-2D*, *Q.Yr.bisa-5B.2*, and *Q.Yr.bisa-7A*) of the eight identified QTLs associated with *Pst* resistance were possibly new QTLs, and identified candidate genes can offer valuable insights on the genetic architecture of the stripe rust disease resistance. CIMMYT advanced wheat lines contain ample known and unknown resistance genes for stripe rust, which could be used to accelerate their validation and deployment in wheat breeding with effective stripe rust resistance. Identification of these genes and QTLs would help in laying a foundation for the biosensor diagnostics techniques, which are displacing conventional detection diagnostics approaches. Due to the small and portable size of biosensors with power of rapid detection, they could aid in precision agriculture applications. Over the next few years, it is anticipated that the new biosensor will reveal many more new insights into the internal workings of plants and their responses to external stimuli.

## Data Availability Statement

The original contributions presented in the study are included in the article/Supplementary Material, further inquiries can be directed to the corresponding author/s.

## Author Contributions

VT wrote the manuscript. VT, DS, GD, and AC analyzed the data. RS provided the breeding material. JP provided genotyping data. VT, PKB, and UK managed field trials and recorded the phenotypic data. VT, GD, DS, UK, AC, RS, JP, and AJ revised the manuscript. UK, RS, JP, and AJ designed the experiment and supervised the research project. All authors have read and approved the final manuscript.

## Conflict of Interest

The authors declare that the research was conducted in the absence of any commercial or financial relationships that could be construed as a potential conflict of interest.

## Publisher’s Note

All claims expressed in this article are solely those of the authors and do not necessarily represent those of their affiliated organizations, or those of the publisher, the editors and the reviewers. Any product that may be evaluated in this article, or claim that may be made by its manufacturer, is not guaranteed or endorsed by the publisher.

## Footnotes

1
https://www.uswheat.org/wheatletter/first-look-at-2019-20-by-usdasees-another-record-world-wheat-crop

## Supplementary Material

Supplementary Figure 1 Distribution of adult plant stage stripe rust severity (%) response based on BLUEs corresponding G × E (E4) environment in 141 advanced breeding lines, where *X*-axis represents the scale of severity (%) and *Y*-axis represent the frequency of ABLs having severity (%).

Supplementary Table 1 List of 141 Genotypes with pedigree information used in present study.

Supplementary Table 2 SNP Hapmap file used in present study.

Supplementary Table 3 Phenotypic data used in present study.

Supplementary Table 4 Avirulence/virulence pattern YR pathotypes used in the study.

Supplementary Table 5 Marker trait associations identified using FarmCPU algorithm for rust disease severity scores across four (individual and G × E) environments.

## References

[B1] Alvarado G. Rodríguez F. M. Pacheco A. Burgueño J. Crossa J. Vargas M. (2020). META-R: a software to analyze data from multi-environment plant breeding trials. *Crop J.* 8 745–756. 10.1016/j.cj.2020.03.010

[B2] Appels R. Eversole K. Feuillet C. Keller B. Rogers J. Stein N. (2018). Shifting the limits in wheat research and breeding using a fully annotated reference genome. *Science* 361:eaar7191. 10.1126/science.aar7191 30115783

[B3] Arora S. Kaur S. Dhillon G. S. Singh R. Kaur J. Sharma A. (2021). Introgression and genetic mapping of leaf rust and stripe rust resistance in *Aegilops triuncialis*. *J. Genet.* 100 1–11. 10.1007/S12041-020-01253-3 33707357

[B4] Azizinia S. Bariana H. Kolmer J. Pasam R. Bhavani S. Chhetri M. (2020). Genomic prediction of rust resistance in tetraploid wheat under field and controlled environment conditions. *Agronomy* 10:1843. 10.3390/agronomy10111843

[B5] Benjamini Y. Hochberg Y. (1995). Controlling the false discovery rate: a practical and powerful approach to multiple testing. *J. R. Stat. Soc. Ser. B* 57 289–300. 10.1111/j.2517-6161.1995.tb02031.x

[B6] Bernardo R. Yu J. (2007). Prospects for genomewide selection for quantitative traits in maize. *Crop Sci.* 47 1082–1090. 10.2135/cropsci2006.11.0690

[B7] Bradbury P. J. Zhang Z. Kroon D. E. Casstevens T. M. Ramdoss Y. Buckler E. S. (2007). TASSEL: software for association mapping of complex traits in diverse samples. *Bioinformatics.* 23 2633–2635. 10.1093/bioinformatics/btm308 17586829

[B8] Breiman L. Cutler A. Wiener M. Liaw Andy. (2001). Package “randomForest” breiman and cutler’s random forests for classification and regression. *Mach. Learn.* 2 18–22.

[B9] Browning B. L. Browning S. R. (2016). Genotype imputation with millions of reference samples. *Am. J. Hum. Genet.* 98 116–126. 10.1016/j.ajhg.2015.11.020 26748515 PMC4716681

[B10] Brueggeman R. Rostoks N. Kudrna D. Kilian A. Han F. Chen J. (2002). The barley stem rust-resistance gene Rpg1 is a novel disease-resistance gene with homology to receptor kinases. *Proc. Natl. Acad. Sci. U.S.A.* 99 9328–9333. 10.1073/pnas.142284999 12077318 PMC123140

[B11] Chao S. Dubcovsky J. Dvorak J. Luo M. C. Baenziger S. P. Matnyazov R. (2010). Population- and genome-specific patterns of linkage disequilibrium and SNP variation in spring and winter wheat (*Triticum aestivum* L.). *BMC Genomics* 11:727. 10.1186/1471-2164-11-727 21190581 PMC3020227

[B12] Charmet G. Tran L. G. Auzanneau J. Rincent R. Bouchet S. (2020). BWGS: a R package for genomic selection and its application to a wheat breeding programme. *PLoS One* 15:e0222733. 10.1371/journal.pone.0222733 32240182 PMC7141418

[B13] Chen X. M. (2005). Epidemiology and control of stripe rust [*Puccinia striiformis f. sp. tritici*] on wheat. *Can. J. Plant Pathol.* 27 314–337. 10.1080/07060660509507230

[B14] Cheng W. Yin S. Tu Y. Mei H. Wang Y. Yang Y. (2020). SlCAND1, encoding cullin-associated Nedd8-dissociated protein 1, regulates plant height, flowering time, seed germination, and root architecture in tomato. *Plant Mol. Biol.* 102 537–551. 10.1007/s11103-020-00963-7 31916084

[B15] Crossa J. De Los Campos G. Pérez P. Gianola D. Burgueño J. Araus J. L. (2010). Prediction of genetic values of quantitative traits in plant breeding using pedigree and molecular markers. *Genetics* 186 713–724. 10.1534/genetics.110.118521 20813882 PMC2954475

[B16] Crossa J. Pérez-Rodríguez P. Cuevas J. Montesinos-López O. Jarquín D. de los Campos G. (2017). Genomic selection in plant breeding: methods, models, and perspectives. *Trends Plant Sci.* 22 961–975. 10.1016/j.tplants.2017.08.011 28965742

[B17] Cui S. Huang F. Wang J. Ma X. Cheng Y. Liu J. (2005). A proteomic analysis of cold stress responses in rice seedlings. *Proteomics* 5 3162–3172. 10.1002/pmic.200401148 16078185

[B18] Daetwyler H. D. Bansal U. K. Bariana H. S. Hayden M. J. Hayes B. J. (2014). Genomic prediction for rust resistance in diverse wheat landraces. *Theor. Appl. Genet.* 127 1795–1803. 10.1007/s00122-014-2341-8 24965887

[B19] Das A. Parihar A. K. Saxena D. Singh D. Singha K. D. Kushwaha K. P. S. (2019). Deciphering genotype-by- Environment interaction for targeting test environments and rust resistant genotypes in field pea (*Pisum sativum* l.). *Front. Plant Sci.* 10:825. 10.3389/fpls.2019.00825 31354749 PMC6635599

[B20] De Los Campos G. Gianola D. Rosa G. J. M. Weigel K. A. Crossa J. (2010). Semi-parametric genomic-enabled prediction of genetic values using reproducing kernel Hilbert spaces methods. *Genet. Res. (Camb)* 92 295–308. 10.1017/S0016672310000285 20943010

[B21] Dhillon G. S. Kaur S. Das N. Singh R. Poland J. Kaur J. (2020). QTL mapping for stripe rust and powdery mildew resistance in *Triticum durum* – *Aegilops speltoides* backcross introgression lines. *Plant Genet. Resour. Characterisation Util.* 18 211–221. 10.1017/S1479262120000222

[B22] Dobritsa A. A. Shrestha J. Morant M. Pinot F. Matsuno M. Swanson R. (2009). CYP704B1 is a long-chain fatty acid ω-Hydroxylase essential for sporopollenin synthesis in pollen of *Arabidopsis*. *Plant Physiol.* 151 574–589. 10.1104/pp.109.144469 19700560 PMC2754625

[B23] Dreisigacker S. Deepmala S. Jaimez R. A. Luna B. G. Muñoz S. Z. Ríos C. N. C. (2016). *CIMMYT Wheat Molecular Genetics: Laboratory Protocols and Applications to Wheat Breeding.* Mexico: CIMMYT.

[B24] Edae E. A. Pumphrey M. O. Rouse M. N. (2018). A genome-wide association study of field and seedling response to individual stem rust pathogen races reveals combinations of race-specific genes in north american spring wheat. *Front. Plant Sci.* 9:52. 10.3389/fpls.2018.00052 29441083 PMC5797647

[B25] Elbasyoni I. S. El-Orabey W. M. Morsy S. Baenziger P. S. Al Ajlouni Z. Dowikat I. (2019). Evaluation of a global spring wheat panel for stripe rust: resistance loci validation and novel resources identification. *PLoS One* 14:e0222755. 10.1371/journal.pone.0222755 31721783 PMC6853611

[B26] Friedman J. Hastie T. Tibshirani R. (2010). Regularization paths for generalized linear models via coordinate descent. *J. Stat. Softw.* 33 1–22. 10.18637/jss.v033.i01 20808728 PMC2929880

[B27] Fu D. Uauy C. Distelfeld A. Blechl A. Epstein L. Chen X. (2009). A kinase-START gene confers temperature-dependent resistance to wheat stripe rust. *Science* 323 1357–1360. 10.1126/science.1166289 19228999 PMC4737487

[B28] Gao L. Kathryn Turner M. Chao S. Kolmer J. Anderson J. A. (2016). Genome wide association study of seedling and adult plant leaf rust resistance in elite spring wheat breeding lines. *PLoS One* 11:e0148671. 10.1371/journal.pone.0148671 26849364 PMC4744023

[B29] Garland M. Schulze C. J. Foe I. T. Van Der Linden W. A. Child M. A. Bogyo M. (2018). Development of an activity-based probe for acyl-protein thioesterases. *PLoS One* 13:e0190255. 10.1371/journal.pone.0190255 29364904 PMC5783350

[B30] Gianola D. Van Kaam J. B. C. H. M. (2008). Reproducing kernel Hilbert spaces regression methods for genomic assisted prediction of quantitative traits. *Genetics* 178 2289–2303. 10.1534/genetics.107.084285 18430950 PMC2323816

[B31] Guan Q. Wu J. Zhang Y. Jiang C. Liu R. Chai C. (2013). A DEAD box RNA helicase is critical for pre-mRNA splicing, cold-responsive gene regulation, and cold tolerance in *Arabidopsis*. *Plant Cell.* 25 342–356. 10.1105/tpc.112.108340 23371945 PMC3584546

[B32] Han F. P. Fedak G. Ouellet T. Dan H. Somers D. J. (2005). Mapping of genes expressed in *Fusarium graminearum*-infected heads of wheat cultivar “Frontana.”. *Genome* 48 88–96. 10.1139/g04-098 15729400

[B33] He F. Ding S. Wang H. Qin F. (2020). IntAssoPlot: an r package for integrated visualization of genome-wide association study results with gene structure and linkage disequilibrium matrix. *Front. Genet.* 11:260. 10.3389/fgene.2020.00260 32265990 PMC7100855

[B34] Heffner E. L. Sorrells M. E. Jannink J. L. (2009). Genomic selection for crop improvement. *Crop Sci.* 49 1–12. 10.2135/cropsci2008.08.0512

[B35] Iwata H. Jannink J. L. (2011). Accuracy of genomic selection prediction in barley breeding programs: a simulation study based on the real single nucleotide polymorphism data of barley breeding lines. *Crop Sci.* 51 1915–1927. 10.2135/cropsci2010.12.0732

[B36] Jannink J. L. Lorenz A. J. Iwata H. (2010). Genomic selection in plant breeding: from theory to practice. *Briefings Funct. Genomics Proteomics* 9 166–177. 10.1093/bfgp/elq001 20156985

[B37] Jiang Y. Reif J. C. (2015). Modeling epistasis in genomic selection. *Genetics* 201 759–768. 10.1534/genetics.115.177907 26219298 PMC4596682

[B38] Jin H. Xu M. Chen H. Zhang S. Han X. Tang Z. (2016). Comparative proteomic analysis of differentially expressed proteins in *Amaranthus hybridus* L. Roots under cadmium stress. *Water Air. Soil Pollut.* 227 1–12. 10.1007/s11270-016-2914-z

[B39] Jiwuba L. Danquah A. Asante I. Blay E. Onyeka J. Danquah E. (2020). Genotype by environment interaction on resistance to cassava green mite associated traits and effects on yield performance of cassava genotypes in Nigeria. *Front. Plant Sci.* 11:572200. 10.3389/fpls.2020.572200 33013995 PMC7498573

[B40] Juliana P. Singh R. P. Singh P. K. Crossa J. Huerta-Espino J. Lan C. (2017). Genomic and pedigree-based prediction for leaf, stem, and stripe rust resistance in wheat. *Theor. Appl. Genet.* 130 1415–1430. 10.1007/s00122-017-2897-1 28393303 PMC5487692

[B41] Juliana P. Singh R. P. Singh P. K. Poland J. A. Bergstrom G. C. Huerta-Espino J. (2018). Genome-wide association mapping for resistance to leaf rust, stripe rust and tan spot in wheat reveals potential candidate genes. *Theor. Appl. Genet.* 131 1405–1422. 10.1007/s00122-018-3086-6 29589041 PMC6004277

[B42] Kankwatsa P. Singh D. Thomson P. C. Babiker E. M. Bonman J. M. Newcomb M. (2017). Characterization and genome-wide association mapping of resistance to leaf rust, stem rust and stripe rust in a geographically diverse collection of spring wheat landraces. *Mol. Breed.* 37:113. 10.1007/s11032-017-0707-8

[B43] Kaur J. Kaur J. Dhillon G. S. Kaur H. Singh J. Bala R. (2021). Characterization and mapping of spot blotch in *Triticum durum*–*Aegilops speltoides* introgression lines using SNP markers. *Front. Plant Sci.* 12:650400. 10.3389/FPLS.2021.650400 34122476 PMC8193842

[B44] Klymiuk V. Yaniv E. Huang L. Raats D. Fatiukha A. Chen S. (2018). Cloning of the wheat Yr15 resistance gene sheds light on the plant tandem kinase-pseudokinase family. *Nat. Commun.* 9:3735. 10.1038/s41467-018-06138-9 30282993 PMC6170490

[B45] Kourelis J. Van Der Hoorn R. A. L. (2018). Defended to the nines: 25 years of resistance gene cloning identifies nine mechanisms for R protein function. *Plant Cell* 30 285–299. 10.1105/tpc.17.00579 29382771 PMC5868693

[B46] Kruger W. M. Pritsch C. Chao S. Muehlbauer G. J. (2002). Functional and comparative bioinformatic analysis of expressed genes from wheat spikes infected with *Fusarium graminearum*. *Mol. Plant-Microbe Interact.* 15 445–455. 10.1094/MPMI.2002.15.5.445 12036275

[B47] La Camera S. Balagué C. Göbel C. Geoffroy P. Legrand M. Feussner I. (2009). The *Arabidopsis* patatin-like protein 2 (PLP2) plays an essential role in cell death execution and differentially affects biosynthesis of oxylipins and resistance to pathogens. *Mol. Plant-Microbe Interact.* 22 469–481. 10.1094/MPMI-22-4-0469 19271961

[B48] Liang X. Zhou J. M. (2018). Receptor-like cytoplasmic kinases: central players in plant receptor kinase-mediated signaling. *Annu. Rev. Plant Biol.* 69 267–299. 10.1146/annurev-arplant-042817-040540 29719165

[B49] Line R. F. Qayoum A. (1992). *Virulence, Aggressiveness, Evolution and Distribution of Races of Puccinia striiformis (the Cause of Stripe of Wheat) in North America, 1968–1987. U.S. Dep. Agric. Tech. Bull. No. 1788.* Rome: FAO.

[B50] Liu W. Maccaferri M. Bulli P. Rynearson S. Tuberosa R. Chen X. (2017). Genome-wide association mapping for seedling and field resistance to *Puccinia striiformis* f. sp. tritici in elite durum wheat. *Theor. Appl. Genet.* 130 649–667. 10.1007/s00122-016-2841-9 28039515

[B51] Lu Y. Lan C. Liang S. Zhou X. Liu D. Zhou G. (2009). QTL mapping for adult-plant resistance to stripe rust in Italian common wheat cultivars *Libellula* and *Strampelli*. *Theor. Appl. Genet.* 119 1349–1359. 10.1007/s00122-009-1139-6 19756474

[B52] Maccaferri M. Zhang J. Bulli P. Abate Z. Chao S. Cantu D. (2015). A genome-wide association study of resistance to stripe rust (*Puccinia striiformis f. sp. tritici*) in a worldwide collection of hexaploid spring wheat (*Triticum aestivum* L.). *G3* 5 449–465. 10.1534/g3.114.014563 25609748 PMC4349098

[B53] Maytalman D. Mert Z. Baykal A. T. Inan C. Günel A. Hasan ebi S. (2013). Proteomic analysis of early responsive resistance proteins of wheat (*Triticum aestivum*) to yellow rust (*Puccinia striiformis f. sp. tritici*) using ProteomeLab PF2D. *Plant Omics* 6 24–35. Available online at: https://search.informit.org/doi/abs/10.3316/informit.226486321658297 (accessed September 6, 2021).

[B54] Meuwissen T. H. E. Hayes B. J. Goddard M. E. (2001). Prediction of total genetic value using genome-wide dense marker maps. *Genetics* 157 1819–1829. 11290733 10.1093/genetics/157.4.1819 PMC1461589

[B55] Muleta K. T. Bulli P. Zhang Z. Chen X. Pumphrey M. (2017). Unlocking diversity in germplasm collections via genomic selection: a case study based on quantitative adult plant resistance to stripe rust in spring wheat. *Plant Genome* 10 1–15. 10.3835/plantgenome2016.12.0124 29293811

[B56] Olukolu B. A. Tracy W. F. Wisser R. De Vries B. Balint-Kurti P. J. (2016). A genome-wide association study for partial resistance to maize common rust. *Phytopathology* 106 745–751. 10.1094/PHYTO-11-15-0305-R 27003507

[B57] Ornella L. Singh S. Perez P. Burgueño J. Singh R. Tapia E. (2012). genomic prediction of genetic values for resistance to wheat rusts. *Plant Genome* 3 136–148. 10.3835/plantgenome2012.07.0017

[B58] Owens B. F. Gore M. A. Magallanes-Lundback M. Tiede T. Diepenbrock C. H. Kandianis C. B. (2014). A foundation for provitamin a biofortification of maize: Genome-wide association and genomic prediction models of carotenoid levels. *Genetics* 198 1699–1716. 10.1534/genetics.114.169979 25258377 PMC4256781

[B59] Parmley K. Nagasubramanian K. Sarkar S. Ganapathysubramanian B. Singh A. K. (2019). Development of optimized phenomic predictors for efficient plant breeding decisions using phenomic-assisted selection in soybean. *Plant Phenomics* 2019:580940. 10.34133/2019/5809404 33313530 PMC7706298

[B60] Poland J. A. Brown P. J. Sorrells M. E. Jannink J. L. (2012). Development of high-density genetic maps for barley and wheat using a novel two-enzyme genotyping-by-sequencing approach. *PLoS One* 7:e32253. 10.1371/journal.pone.0032253 22389690 PMC3289635

[B61] Pure G. A. Chakravorty A. K. Scott K. J. (1984). Changes in the template activity of chromatin and the transcriptive specificity of RNA polymerase II of wheat leaves during the early stages of rust infection. *Physiol. Plant Pathol.* 25 71–82. 10.1016/0048-4059(84)90018-3

[B62] R Core Team. (2019). *R: A Language and Environment for Statistical Computing. R Found. Stat. Comput.* Vienna: R Core Team.

[B63] Robertsen C. D. Hjortshøj R. L. Janss L. L. (2019). Genomic selection in cereal breeding. *Agronomy* 9:95. 10.3390/agronomy9020095

[B64] Rutkoski J. Singh R. P. Huerta-Espino J. Bhavani S. Poland J. Jannink J. L. (2015a). Efficient use of historical data for genomic selection: a case study of stem rust resistance in wheat. *Plant Genome* 8 1–10. 10.3835/plantgenome2014.09.0046 33228293

[B65] Rutkoski J. Singh R. P. Huerta-Espino J. Bhavani S. Poland J. Jannink J. L. (2015b). Genetic gain from phenotypic and genomic selection for quantitative resistance to stem rust of wheat. *Plant Genome* 8 1–10. 10.3835/plantgenome2014.10.0074 33228306

[B66] Rutkoski J. E. Heffner E. L. Sorrells M. E. (2011). Genomic selection for durable stem rust resistance in wheat. *Euphytica.* 179 161–173. 10.1007/s10681-010-0301-1

[B67] Rutkoski J. E. Poland J. A. Singh R. P. Huerta-Espino J. Bhavani S. Barbier H. (2014). Genomic selection for quantitative adult plant stem rust resistance in wheat. *Plant Genome* 7 1–10. 10.3835/plantgenome2014.02.0006

[B68] Silar P. Picard M. (1994). Increased longevity of EF-1α high-fidelity mutants in *Podospora anserina*. *J. Mol. Biol.* 235 231–236. 10.1016/S0022-2836(05)80029-4 8289244

[B69] Singh H. Kaur J. Bala R. Srivastava P. Bains N. S. (2020). Virulence and genetic diversity of *Puccinia striiformis* f. sp. tritici isolates in sub-mountainous area of Punjab. *India Phytoparasit.* 48 383–395. 10.1007/S12600-020-00809-4

[B70] Smith H. M. S. Hicks G. R. Raikhel N. V. (1997). Importin α from *Arabidopsis thaliana* is a nuclear import receptor that recognizes three classes of import signals. *Plant Physiol.* 114 411–417. 10.1104/pp.114.2.411 9193081 PMC158320

[B71] Strohm A. K. Vaughn L. M. Masson P. H. (2015). Natural variation in the expression of ORGANIC CATION TRANSPORTER 1 affects root length responses to cadaverine in *Arabidopsis*. *J. Exp. Bot.* 66 853–862. 10.1093/jxb/eru444 25403917 PMC4321547

[B72] Tekalign A. Sibiya J. Derera J. Fikre A. (2017). Analysis of genotype × environment interaction and stability for grain yield and chocolate spot (*Botrytis fabae*) disease resistance in faba bean (*Vicia faba*). *Aust. J. Crop Sci.* 11 1228–1235. 10.21475/ajcs.17.11.10.pne413

[B73] Tomar V. Singh D. Dhillon G. S. Singh R. P. Poland J. Joshi A. K. (2021). New QTLs for spot blotch disease resistance in wheat (*Triticum aestivum* L.) using genome-wide association mapping. *Front. Genet.* 11:613217. 10.3389/FGENE.2020.613217 33519916 PMC7841440

[B74] VanRaden P. M. (2008). Efficient methods to compute genomic predictions. *J. Dairy Sci.* 91 4414–4423. 10.3168/JDS.2007-0980 18946147

[B75] Wang J. Zhang Z. (2021). Boosting power and accuracy for genomic association and prediction. *Genom. Proteom. Bioinform.* 10.1016/j.gpb.2021.08.005 (In press). 34492338 PMC9121400

[B76] Wang X. Xu Y. Hu Z. Xu C. (2018). Genomic selection methods for crop improvement: current status and prospects. *Crop J.* 6 330–340. 10.1016/j.cj.2018.03.001

[B77] Watson A. Ghosh S. Williams M. J. Cuddy W. S. Simmonds J. Rey M. D. (2018). Speed breeding is a powerful tool to accelerate crop research and breeding. *Nat. Plants.* 4 23–29. 10.1038/s41477-017-0083-8 29292376

[B78] Xia Y. Suzuki H. Borevitz J. Blount J. Guo Z. Patel K. (2004). An extracellular aspartic protease functions in *Arabidopsis* disease resistance signaling. *EMBO J.* 23 980–988. 10.1038/sj.emboj.7600086 14765119 PMC380998

[B79] Xie L. He X. Shang S. Zheng W. Liu W. Zhang G. (2014). Comparative proteomic analysis of two tobacco (*Nicotiana tabacum*) genotypes differing in Cd tolerance. *BioMetals* 27 1277–1289. 10.1007/s10534-014-9789-5 25173101

[B80] Yang H. Li C. Lam H. M. Clements J. Yan G. Zhao S. (2015). Sequencing consolidates molecular markers with plant breeding practice. *Theor. Appl. Genet.* 128 779–795. 10.1007/s00122-015-2499-8 25821196

[B81] Zadoks J. C. Chang T. T. Konzak C. F. (1974). A decimal code for the growth stages of cereals. *Weed Res.* 14 415–421. 10.1111/j.1365-3180.1974.tb01084.x

[B82] Zhang C. Huang L. Zhang H. Hao Q. Lyu B. Wang M. (2019). An ancestral NB-LRR with duplicated 3’UTRs confers stripe rust resistance in wheat and barley. *Nat. Commun.* 10:4023. 10.1038/s41467-019-11872-9 31492844 PMC6731223

[B83] Zhao Y. Huang J. Wang Z. Jing S. Wang Y. Ouyang Y. (2016). Allelic diversity in an NLR gene BPH9 enables rice to combat planthopper variation. *Proc. Natl. Acad. Sci. U.S.A.* 113 12850–12855. 10.1073/pnas.1614862113 27791169 PMC5111712

