# Supplementary figures and images for: Evaluations of Genomic Prediction and Identification of New Loci for Resistance to Stripe Rust Disease in Wheat (Triticum aestivum L.)
Source: Front Genet. 2021 Sep 28;12:710485. doi: 10.3389/fgene.2021.710485 (PMC8505882; doi:10.3389/fgene.2021.710485)

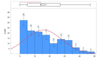

Supplement: Supplementary Figure 1 — Distribution of adult plant stage stripe rust severity (%) response based on BLUEs corresponding G × E (E4) environment in 141 advanced breeding lines, where X-axis represents the scale of severity (%) and Y-axis represent the frequency of ABLs having severity (%). [file Image_1.JPEG]
