# Supplementary material for: Evaluations of Genomic Prediction and Identification of New Loci for Resistance to Stripe Rust Disease in Wheat (Triticum aestivum L.)
Source: Front Genet. 2021 Sep 28;12:710485. doi: 10.3389/fgene.2021.710485 (PMC8505882; doi:10.3389/fgene.2021.710485)
Supplement: Supplementary Table 4 — Avirulence/virulence pattern YR pathotypes used in the study. [file Table_4.DOCX]

**Table S4:** Avirulence/virulence pattern YR pathotypes used in the study.

| Race | Avirulence formula | Virulence formula |
| --- | --- | --- |
| 78S84 | *Yr1, Yr 3b, Yr4b, Yr5, Yr10, Yr11, Yr14, Yr15, Yr17, Yr18, Yr24/26, Yr28, Yr29, YrSd* | *Yr2, Yr3a, Yr4A, Yr6, Yr7, Yr8, Yr9, Yr12, Yr19, YrSk, YrSu, Yr31* |
| 46S119 | *Yr1, Yr5, Yr10, Yr14, Yr15, Yr24, Yr26, Yr28, YrSp* | *Yr2, Yr3a, Yr3b, Yr4a, Yr4b, Yr6, Yr7, Yr8, Yr9, Yr11, Yr12, Yr 17, Yr19, Yr29, Yr31, YrSk* |
| 110S119 | *Yr1, Yr5, Yr10, Yr15, Yr24, Yr26, Yr28, YrSp, Riebesel 147/51(Yr2,9,?)* | *Yr2, Yr3a, Yr3b, Yr4a, Yr4b, Yr6, Yr7, Yr8, Yr9, Yr11, Yr12, Yr14, Yr 17, Yr18, Yr19, Yr29, Yr31, YrSk* |
| 238S119 | *Yr1, Yr5, Yr10, Yr24/ Yr26,* | *Yr2, Yr2+, Yr3a, Yr3b, Yr4a, Yr4a+, Yr4b, Yr6, Yr7, Yr8, Yr9, Yr9+, Yr22, Yr23, YrSd, YrSu* |
