# Supplementary material for: Evaluations of Genomic Prediction and Identification of New Loci for Resistance to Stripe Rust Disease in Wheat (Triticum aestivum L.)
Source: Front Genet. 2021 Sep 28;12:710485. doi: 10.3389/fgene.2021.710485 (PMC8505882; doi:10.3389/fgene.2021.710485)
Supplement: Supplementary Table 5 — Marker trait associations identified using FarmCPU algorithm for rust disease severity scores across four (individual and G × E) environments. [file Table_5.docx]

Table S5: Marker trait associations identified using FarmCPU algorithm for rust disease severity scores across four environments

| SNP | Env | Chr | Pos (Mb) | Pvalue  (-log10) | maf | effect |
| --- | --- | --- | --- | --- | --- | --- |
| S2A_753261678 | E4 | 2A | 753.262 | 12.655 | 0.421986 | -5.52568 |
| S2A_719332307 | E4 | 2A | 719.332 | 11.225 | 0.163121 | -5.49096 |
| S7A_717077502 | E4 | 7A | 717.078 | 6.608 | 0.407801 | 3.728229 |
| S2D_76635286 | E4 | 2D | 76.635 | 5.560 | 0.301418 | 3.517772 |
| S5B_56640410 | E4 | 5B | 56.640 | 5.339 | 0.124113 | -3.59503 |
| S2A_451543170 | E4 | 2A | 451.543 | 5.026 | 0.099291 | 5.114136 |
| S6A_602916015 | E4 | 6A | 602.916 | 4.807 | 0.351064 | -2.74191 |
| S5B_598915625 | E4 | 5B | 598.916 | 4.765 | 0.212766 | 3.039017 |
| S2B_53460517 | E1 | 2B | 53.461 | 3.692 | 0.113475 | 4.319771 |
| S2A_753268026 | E2 | 2A | 753.268 | 3.592 | 0.41844 | -10.1215 |
| S2A_753382980 | E2 | 2A | 753.383 | 3.592 | 0.41844 | -10.1215 |
| S2A_753384128 | E2 | 2A | 753.384 | 3.592 | 0.41844 | 10.12151 |
| S2A_753466912 | E2 | 2A | 753.467 | 3.592 | 0.41844 | 10.12151 |
| S2A_753691877 | E2 | 2A | 753.692 | 3.583 | 0.421986 | 9.99611 |
| S2A_753261678 | E2 | 2A | 753.262 | 3.583 | 0.421986 | -9.99611 |
| S5B_598915625 | E2 | 5B | 598.916 | 3.581 | 0.212766 | 11.57456 |
| S2B_161419325 | E2 | 2B | 161.419 | 3.566 | 0.163121 | -13.1677 |
| S4A_568912343 | E4 | 4A | 568.912 | 3.439 | 0.301418 | -2.72716 |
| S2B_182926961 | E3 | 2B | 182.927 | 3.380 | 0.319149 | -4.09963 |
| S5B_600131111 | E3 | 5B | 600.131 | 3.343 | 0.248227 | -4.2011 |
| S5B_603881915 | E3 | 5B | 603.882 | 3.336 | 0.297872 | 3.96979 |
| S2B_545874061 | E2 | 2B | 545.874 | 3.320 | 0.170213 | 12.403 |
| S2B_546015141 | E2 | 2B | 546.015 | 3.320 | 0.170213 | 12.403 |
| S2A_719332307 | E1 | 2A | 719.332 | 3.318 | 0.163121 | -3.41996 |
| S5B_598117789 | E3 | 5B | 598.118 | 3.301 | 0.241135 | -4.20391 |
| S2B_155142247 | E2 | 2B | 155.142 | 3.300 | 0.156028 | 12.81899 |
| S2B_157110560 | E2 | 2B | 157.111 | 3.300 | 0.156028 | 12.81899 |
| S2B_157114708 | E2 | 2B | 157.115 | 3.300 | 0.156028 | 12.81899 |
| S2B_157152072 | E2 | 2B | 157.152 | 3.300 | 0.156028 | 12.81899 |
| S2B_155155706 | E2 | 2B | 155.156 | 3.300 | 0.156028 | -12.819 |
| S2B_155574746 | E2 | 2B | 155.575 | 3.300 | 0.156028 | -12.819 |
| S2B_156429524 | E2 | 2B | 156.430 | 3.300 | 0.156028 | -12.819 |
| S2B_157266347 | E2 | 2B | 157.266 | 3.300 | 0.156028 | -12.819 |
| S2B_158359468 | E2 | 2B | 158.359 | 3.268 | 0.460993 | -8.42742 |
| S2B_158649503 | E2 | 2B | 158.650 | 3.268 | 0.460993 | -8.42742 |
| S2A_690962587 | E1 | 2A | 690.963 | 3.259 | 0.060284 | 5.362563 |
| S2A_690962408 | E1 | 2A | 690.962 | 3.259 | 0.060284 | -5.36256 |
| S2A_744374225 | E2 | 2A | 744.374 | 3.242 | 0.287234 | 10.21827 |
| S2A_744387449 | E2 | 2A | 744.387 | 3.242 | 0.287234 | 10.21827 |
| S2A_744407030 | E2 | 2A | 744.407 | 3.242 | 0.287234 | 10.21827 |
| S2A_744366488 | E2 | 2A | 744.366 | 3.242 | 0.287234 | -10.2183 |
| S2A_744593454 | E2 | 2A | 744.593 | 3.242 | 0.287234 | -10.2183 |
| S2B_154909145 | E2 | 2B | 154.909 | 3.239 | 0.5 | 8.790705 |
| S2B_157809394 | E2 | 2B | 157.809 | 3.239 | 0.5 | 8.790705 |
| S5B_598915625 | E3 | 5B | 598.916 | 3.231 | 0.212766 | 4.493091 |
| S4D_459685755 | E1 | 4D | 459.686 | 3.212 | 0.049645 | -5.64671 |
| S2A_690330323 | E1 | 2A | 690.330 | 3.200 | 0.053191 | 5.721468 |
| S2A_690201759 | E1 | 2A | 690.202 | 3.200 | 0.053191 | -5.72147 |
| S2A_693269853 | E1 | 2A | 693.270 | 3.200 | 0.053191 | -5.72147 |
| S5B_603879101 | E3 | 5B | 603.879 | 3.178 | 0.269504 | 4.124427 |
| S2B_178771120 | E2 | 2B | 178.771 | 3.171 | 0.319149 | 9.604921 |
| S2A_744408504 | E2 | 2A | 744.409 | 3.124 | 0.283688 | -10.036 |
| S4B_649729222 | E1 | 4B | 649.729 | 3.107 | 0.163121 | -3.30369 |
| S4B_650599974 | E1 | 4B | 650.600 | 3.107 | 0.163121 | 3.30369 |
| S3B_730286179 | E1 | 3B | 730.286 | 3.095 | 0.053191 | 5.513306 |
| S7B_595291938 | E4 | 7B | 595.292 | 3.071 | 0.478723 | 2.159114 |
| S7B_595524174 | E4 | 7B | 595.524 | 3.071 | 0.478723 | 2.159114 |
| S3B_691511687 | E1 | 3B | 691.512 | 3.067 | 0.049645 | 5.569493 |
| S3B_691610833 | E1 | 3B | 691.611 | 3.067 | 0.049645 | 5.569493 |
| S3B_694987123 | E1 | 3B | 694.987 | 3.067 | 0.049645 | 5.569493 |
| S3B_696269501 | E1 | 3B | 696.270 | 3.067 | 0.049645 | 5.569493 |
| S3B_694987152 | E1 | 3B | 694.987 | 3.067 | 0.049645 | -5.56949 |
| S3B_696329092 | E1 | 3B | 696.329 | 3.067 | 0.049645 | -5.56949 |
| S3B_697172580 | E1 | 3B | 697.173 | 3.067 | 0.049645 | -5.56949 |
| S3B_700044002 | E1 | 3B | 700.044 | 3.067 | 0.049645 | -5.56949 |
| S3B_700115096 | E1 | 3B | 700.115 | 3.067 | 0.049645 | -5.56949 |
| S3B_701317234 | E1 | 3B | 701.317 | 3.067 | 0.049645 | -5.56949 |
| S2B_182836231 | E2 | 2B | 182.836 | 3.038 | 0.29078 | -9.63713 |
| S2B_697586255 | E4 | 2B | 697.586 | 3.033 | 0.06383 | -4.59823 |
| S2B_185013224 | E3 | 2B | 185.013 | 3.030 | 0.312057 | 3.929527 |
| S2B_185125024 | E3 | 2B | 185.125 | 3.030 | 0.312057 | 3.929527 |
| S2B_129705054 | E1 | 2B | 129.705 | 3.017 | 0.393617 | -2.5049 |
| S2B_130028901 | E1 | 2B | 130.029 | 3.017 | 0.393617 | 2.504904 |
| S2B_131108414 | E1 | 2B | 131.108 | 3.017 | 0.393617 | -2.5049 |
| S2B_697888874 | E4 | 2B | 697.889 | 3.015 | 0.060284 | -4.61238 |
| S2B_165579143 | E1 | 2B | 165.579 | 3.005 | 0.404255 | -2.52859 |
